# Ocular Surface Temperature: Characterization in a Large Cohort of Healthy Human Eyes and Correlations to Systemic Cardiovascular Risk Factors

**DOI:** 10.3390/diagnostics11101877

**Published:** 2021-10-12

**Authors:** Gal Yaakov Cohen, Gil Ben-David, Reut Singer, Sahar Benyosef, Rachel Shemesh, Ari Leshno, Yaniv Barkana, Alon Skaat

**Affiliations:** 1 The Sam Rothberg Glaucoma Research Center, Goldschleger Eye Institute, Sheba Medical Center, Tel Hashomer, Ramat Gan 5262000, Israel; gal.cohen@sheba.gov.il (G.Y.C.); reutsinger@gmail.com (R.S.); shaharbenyo@gmail.com (S.B.); shemeshrach@gmail.com (R.S.); arileshno@gmail.com (A.L.); alon.skaat@sheba.health.gov.il (A.S.); 2 The Sackler Faculty of Medicine, Tel Aviv University, Tel Aviv 6997801, Israel; gil8000@gmail.com; 3 The Department of Ophthalmology, Rabin Medical Center, Petah Tikva 49100, Israel; 4 The Sheba Talpiot Medical Leadership Program, Sheba Medical Center, Tel Hashomer, Ramat Gan 52621, Israel

**Keywords:** ocular surface temperature, infrared thermography, ischemic heart disease

## Abstract

Purpose: To characterize ocular surface temperature (OST) in healthy eyes and its association with systemic risk factors of cardiovascular and ischemic heart disease. Methods: This prospective cross-sectional study included consenting subjects who were examined at the Institute for Medical Screening in Sheba Medical Center. A Therm-App™ thermal imaging camera (Opgal LTD, Israel) was used for OST acquisition, and the mean OST of the medial canthal, lateral canthal, and central cornea regions were measured. Room and body temperatures were also recorded. Past medical and ocular history as well as data from various clinical examinations performed at the same visit were obtained. Results: Thermographic images were obtained from 186 subjects, 150 of which were included in the final analysis. OST was significantly higher in the medial canthal, central cornea, and lateral canthal regions in people with a history of ischemic heart disease (*p* = 0.02, *p* = 0.02, and *p* = 0.03, respectively). There were no significant OST differences (ANOVA test) associated with the presence of hypertension, diabetes mellitus, or active smoking status. Conclusions: OST correlated positively with the presence of ischemic heart disease. This correlation, its pathophysiological base, and its clinical application warrants further investigation.

## 1. Introduction

Infrared thermography is a noninvasive method that measures infrared radiation emanating from a body’s surface and converts it to temperature readings [1]. Its application in the medical field relies on the effect of pathophysiological processes on surface temperature. For example, it is assumed that inflammatory conditions are associated with a temperature rise, whereas ischemic and vascular disorders are assumed to cause relative tissue cooling [2,3]. Recent technological improvements have produced images with sufficiently high resolution that enable the use of this technique in the clinical setting in order to detect and define vascular, inflammatory, and neoplastic pathologies [4,5,6]. It is currently applied in the diagnosis of rheumatic diseases, vascular occlusive disorders, tissue viability, and oncological and dermatological pathologies [4].

Ocular surface temperature (OST), which is measured on the anterior surface of the eye globe, is determined by a combination of ocular hemodynamics, core body temperature, tear film features, and environment influences, and it can be evaluated by means of infrared thermography [7,8,9,10,11]. There are abundant data on the correlation between OST and changes of both the anterior and posterior segments in a variety of ophthalmic pathologies, such as dry eye syndrome, anterior uveitis, central retinal vein occlusion, diabetic retinopathy, and glaucoma [7,12,13,14,15,16,17,18]. In contrast, there is a lack of data on the effect of systemic disease on OST and only limited data on OST in healthy eyes [19,20,21,22,23,24].

Previous publications have demonstrated that systemic conditions which impair peripheral perfusion are associated with a decrease in OST [24,25]. We designed the current study to explore possible effects of cardiovascular disease on OST. Our purpose was to utilize our access to a large cohort of individuals undergoing both ocular and medical screening examinations that included cardiovascular assessments in order to collect OST measurements of healthy eyes and to identify possible correlations between selected cardiovascular risk factors, ischemic heart disease, and OST findings.

## 2. Methods

### 2.1. Study Design and Study Population

This is a cross-sectional study of individuals who had been referred to undergo an annual medical screening evaluation at the Sheba Institute for Medical Screening between June and December 2020. The institute provides medical screening services for individuals over 40 years of age. The study followed the tenets of the Declaration of Helsinki and was approved by the Institutional Review Board at Sheba Medical Center. Informed consent was obtained from each study participant upon admission, after an explanation of the purpose of the study and description of its procedures. Subjects were suitable for enrollment if they reported neither current nor past eye disease nor prior eye surgery, other than an uneventful cataract surgery at least 3 months prior to the current examination.

Each individual then underwent the routine series of examinations, which included a measurement of vital signs, a complete medical history, laboratory tests, a cardiovascular examination, a physical examination by an internal medicine specialist, and a comprehensive ocular examination by an ophthalmologist. Past medical history data were based upon the anamnesis taken during the index visit as well as upon data collected during previous visits to the institute for medical screening and stored in the institute’s electronic medical records (EMR). Blood tests consisted of a complete blood count, chemistry panel, lipid profile, and thyroid function tests. The cardiovascular physical examination was carried out by a cardiologist, and followed by a standard cardiac stress test according to the Bruce protocol. The ocular examination included biomicroscopy with dilated funduscopy and intraocular pressure (IOP) measurement by means of a puff tonometer (Huvitzs, Gunpo, South Korea). Subjects found to have any eye pathology were excluded.

### 2.2. Thermographic Image Capture

**Figure 1:**
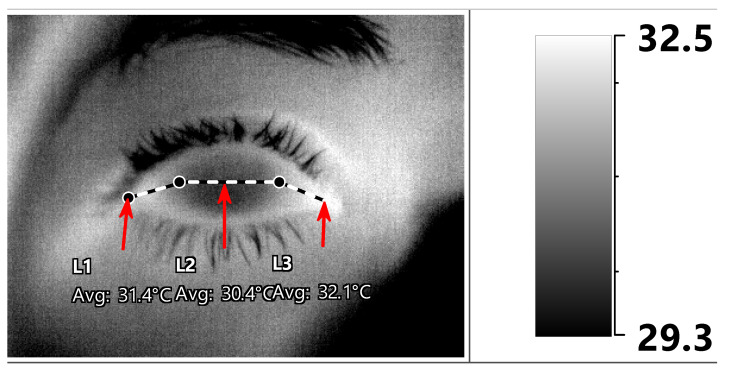
Post-acquisition ocular surface temperature (OST) manual measurements. The mean OST was measured along three horizontal lines (medial cantus -> nasal limbus -> temporal limbus -> lateral cantus) designated as medial canthal, central corneal, and lateral canthal ocular surface regions (L1, L2, and L3, respectively, indicated by red arrows).

Thermography was performed in a designated room as an additional exam for the purposes of this study during the same visit. The entire session was documented in the institute’s EMR from which the data were subsequently extracted for analysis. The same testing room was used throughout the study. It was windowless, artificially lit, and cooled by a central air-conditioning system with a constant setting. Room temperature and humidity, as well as oral core body temperature, were measured and recorded prior to image capture. OST measurements were performed by one of two authors (GC, GBD) using a Therm-App^®^ Pro TH camera (Opgal LTD, Karmiel, Israel) with a 9-mm lens (384*288 pixel resolution; see Appendix A for further specifications) which was operated with a cord connection to a Mi Note 3 smartphone (Xiaomi, Beijing, China). Prior to undergoing the thermographic exam, the subjects were asked to stay in the test room for 20 min in order to achieve temperature acclimatization. They were also requested to keep their eyes closed for 10 s prior to image capture in order to decrease the influence of tear-film evaporation, and to fixate on the center of the camera’s lens. An image capturing the eye surface temperature (Figure 1) was obtained immediately upon eyelid opening.

### 2.3. OST Measurements

The OST was then retrieved from the thermographic images with IRT Cronista^®^ 4.0 software (GRAYESS Inc. 1903 60th Place, Bradenton, FL, USA). We followed the methodology of previous publications as well as that derived from our own observations of a significant difference in OST measurements of various ocular surface regions, and that a measurement of a single area should not be considered as a reference for the average OST [24,25]. Both manual and automated methods of post-acquisition OST measurements have been described [25]. We used a manual procedure in which the mean OST was measured along three horizontal lines representing three ocular surface regions: (1) between the medial cantus and the nasal limbus; (2) between the nasal and temporal limbus; and (3) between the temporal limbus and lateral cantus (Figure 1). All measurements were carried out by a single ophthalmologist (G.C). In addition, image quality was evaluated by the same physician, and it was determined as being “adequate” if anatomical landmarks, such as the corneal limbus and the plica semilunaris, could be easily identified.

### 2.4. Statistical Analysis

Subject characteristics and the prevalence of cardiovascular risk factors were summarized by descriptive statistics. Continuous variables are shown as mean ± standard deviation (SD) and categorical data as frequency and percentage. The Kolmogorov-Smirnov test was used to examine the distribution of continuous variables. We used the GLM repeated measures ANOVA with two factors (examined the left or right eye and the ocular surface region) in order to detect differences between eyes as well as between the regions. Pearson’s correlations coefficients were calculated for continuous variables and the one-way ANOVA test for categorical variables to analyze the effect of demographic and clinical variables on OST. Linear regression models, stepwise methods, were then performed for a multivariate analysis, which included the factors that had been found to be significantly related to OST in the univariate analysis. The statistical software SPSS version 27.0 (SPSS Inc., Chicago, IL, USA) was used for data analysis. Statistical significance was set at *p* < 0.05.

## 3. Results

A total of 186 subjects were initially enrolled, 36 of whom were excluded (19 due to signs of blepharitis, 9 due to a history of corneal refractive surgery, 5 due to either pterygium on examination or a history of pterygium excision, 1 due to a history of strabismus surgery, 1 due to glaucoma with active topical treatment, and 1 due to a history of diabetic retinopathy). The mean ± SD age of the 150 study participants was 52.3 ± 10.8; 93 subjects (62%) were males and 57 (38%) were females. Table 1 describes the subjects’ demographic and clinical characteristics together with the laboratory results. The thermographic images were all graded as “adequate”.

**Figure 2:**
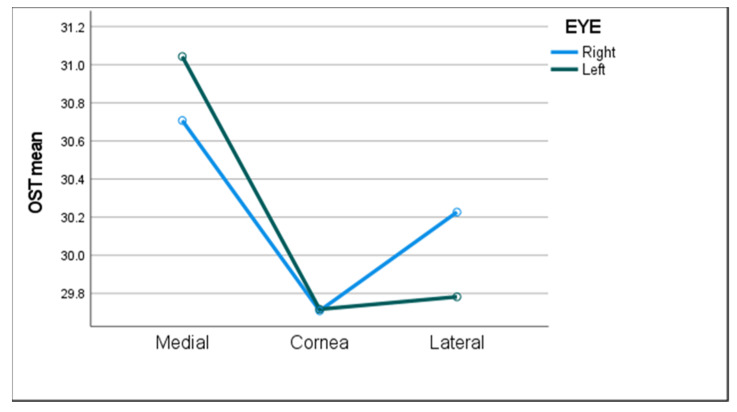
The mean ocular surface temperature (OST) in all three ocular surface regions of both eyes.

Descriptive statistics of OST measurements in all subjects in both eyes along the different ocular surface regions are presented in Table 2. There was a significant difference in OST between ocular surface regions, with the mean temperature at the medial canthal region being the highest (1–1.2 °C higher than the central cornea and 0.5–1.2 °C higher than the lateral canthal region, Figure 2). There was no significant difference between the two eyes (*p* = 0.4), and the left eye was randomly selected in the statistical analysis and group comparisons shown below.

The univariate analysis yielded a significant sex difference in OST in the central cornea, with values 0.5 °C higher in males (*p* = 0.01). OST was also significantly higher in all three regions in the eyes of subjects who had a history of ischemic heart disease (IHD) (*p* < 0.03 for each). There was no significant OST difference associated with hypertension, diabetes mellitus, active smoking status, or obstructive sleep apnea, nor with a pathologic result on the cardiac stress test (Table 3).

OST significantly correlated with low-density lipoprotein levels (LDL) and maximal heart rate in the ergometric cardiac stress test, while it correlated negatively with age and high density lipoprotein (HDL) levels (Table 4).

The measured room temperature was 21.7 °C ± 1.6 and the average humidity was 56.4% ± 11. Statistically significant low-to-medium positive correlations were found between OST and those environmental factors, and those correlations were observed in all three ocular surface regions.

Factors with a significant correlation to OST (both continuous and categorical) were included in a multivariate analysis to predict OST (Table 5). A history of IHD, room temperature variables, and the maximal heart rate as measured during the cardiac stress test were significant predictors of OST in all three ocular surface regions. LDL levels and body temperature were significant predictors of OST in the medial and lateral canthal regions.

## 4. Discussion

In the current study, we characterized the OST in healthy eyes and its association with systemic risk factors of cardiovascular and ischemic heart disease. Our results demonstrated a direct association between ischemic heart disease and OST. Male sex was also associated with a higher OST although this difference was statistially significant only at the central corneal region, and sex did not remain a significant predictor of OST in the multivariate analysis.

As demonstrated by others [20,24], we did not find any significant difference in OST between the right and left eyes of the same individual. We did, however, observe a significant difference in OST between the different ocular regions; the highest being at the medial canthal region and lowest at the central corneal region. We consider that this observed thermal distribution is due to perfusion differences between the avascular cornea and the conjunctiva with its rich vascular supply. These findings are in line with the current body of evidence on OST [8,9,10,19,24].

The observed strong effects of room and body temperature on OST have been consistently described and they were found to be positively linked to OST [9,19,23,24]. In our cohort, the positive correlation to room temperature was significant in all three regions, and the correlation to body temperature was significant at the medial and lateral canthal region. The standard deviation for room temperature was 1.6 °C, probably due to the fact that our measurements were conducted in a clinical environment over a period of six months (summer and winter). This represented a large deviation; however, it is comparable to that of other similar publications [16,26]. While tighter control of room temperature is beneficial for future research, measurements in the clinical setting are inevitably prone to some degree of range, indicating that OST measurements must be adjusted for room temperature.

Ischemic conditions are a well-known cause of decreased tissue temperature as a result of poor tissue perfusion. Thermography has been used to demonstrate this association between ischemic conditions and decreased OST for a number of clinical applications. Chatchawan et al. reported a positive correlation between blood flow and foot skin temperature in type 2 diabetes mellitus patients [27]. Spence et al. concluded that thermography is a reliable method for detecting limb viability before amputation [28]. The correlation between surface temperature and perfusion was also demonstrated in relation to the human eye in both experimental and clinical studies. Gallasi et al. and others have used color Doppler ultrasound coupled with thermography of the ocular surface to directly demonstrate how increased blood flow in the periorbital and ocular vasculature is associated with increased OST and vice versa [29].

Effects on the ocular circulation on OST were also demonstrated in various systemic conditions. In our previous publication regarding OST in retinal disease, a significant decrease in OST was demonstrated among patients with diabetic retinopathy [30]. Morgan et al. reported a decrease in OST among patients with unilateral carotid artery stenosis in the ipsilateral eye [31]. These findings suggest that both macro and microvascular changes in vessels supplying the eye can be evident on OST measurements. In the present work, diabetes, hypertension, and reported active smoking were not significant predictors of OST, while dyslipidemia seemed to have a stronger effect. Patients who reported having dyslipidemia had higher OST values with borderline significance in all three regions. The observed effect was stronger when the analysis was based upon LDL and HDL levels and remained significant during the multivariate analysis.

LDL is a large molecule comprised of many proteins as well as lipids, including cholesterol, phospholipids, and triglycerides. Circulating levels of LDL are directly associated with atherosclerosis disease severity. Once thought to simply be caused by passive retention of LDL in the vasculature, atherosclerosis studies over the past 40–50 years have uncovered a much more complex mechanism. It has now become well established that LDL can undergo many different types of oxidative modifications within the vasculature, such as esterification and lipid peroxidation. The resulting oxidized LDL has been found to have antigenic potential and to contribute considerably to atherosclerosis-associated inflammation, activating both innate and adaptive immunity [32]. We were surprised to see that LDL levels were positively linked to OST, while HDL levels had the opposite effect. Given that LDL and HDL levels are consistently described as key markers for atherosclerosis and comprise a main target of treatment in managing patients with cardiovascular disease, we expected to observe lower OST readings among subjects with high LDL levels, representing reduced tissue perfusion.

We were even more surprised by the significant increase in OST among individuals with a history of ischemic heart disease. One possible explanation relates to the inflammatory nature of atherosclerosis that has been established in several recent investigations. Our observation could be partially explained if the inflammatory nature of atherosclerosis would have a larger effect than the perfusion deficit it causes. Another possible explanation is the use of vasodilating agents, such as beta and calcium channel blockers, by patients with IHD. Our observations merit further investigations specifically designed to improve our understanding of these relationships.

Our work has several limitations. Although we presented one of the largest cohorts to date, the subgroup analyzed for each factor was fairly small and, therefore, our study is underpowered to demonstrate additional significant links of cardiovascular risk factors and OST. Tighter control of environmental factors, such as room temperature, may help improve our measurements and lead to more significant results. While relatively extensive data were collected for each patient, there are several parameters which were not taken into account, such as ethnicity or country of origin, current prescribed medication, and family history. The analysis of the thermographic images was also limited by the software available to us.

In conclusion, we have described the measurements of OST in healthy eyes as they relate to cardiovascular disorders. Our findings indicate that OST may be correlated to IHD. This correlation, its pathophysiological base, and its clinical application should be further investigated in order to evaluate its potential use as a clinical screening test. Our results also encourage further investigation regarding the association between cardiac function and OST, as well as the possible use of OST as a biomarker for IHD.

## Figures and Tables

**Table 1:** Demographic and clinical characteristics and laboratory findings.

Demographic Characteristics	Variable	
	Age (year)	52.3 ± 10.8
	Male sex, *n*	93 (62%)
Medical history, *n*		
	Physically active	118 (85.5%)
	HTN	22 (15.2%)
	DM	12 (8.3%)
	Anemia	10 (6.9%)
	Dyslipidemia	73 (50.3%)
	Active smoker	25 (17.4%)
	OSA	5 (3.4%)
	IHD	9 (6.2%)
	Previous CVA/TIA	2 (1.4%)
	Any past medical history	78 (52.3%)
	Any past ocular history	25 (17%)
Physical examination		
	BMI	26.1 ± 4.0
	Systolic BP (mmHg)	125.0 ± 18.2
	Diastolic BP (mmHg)	75.7 ± 10.3
	HR (bpm)	69.4 ± 14.6
	Body temperature (°C)	36.6 ± 0.2
Cardiac stress test (CST)		
	Normal CST, *n*	124 (82.7%)
	METS	12.0 ± 3.2
	Recovery time (min)	4.7 ± 1.2
	Max HR in ergometry (bpm)	159.4 ± 15.9
	Max systolic BP (mmHg)	167.0 ± 20.7
	Max diastolic BP (mmHg)	77.3 ± 8.0
Laboratory findings		
	Hb (gr/dl)	14.2 ± 1.2
	WBC (cells/mm^3^)	6.3 ± 1.5
	Creatinine (mg/dL)	0.9 ± 0.2
	AST (IU/L)	22.8 ± 7.6
	ALT(IU/L)	21.5 ± 12.0
	TSH (mIU/L)	2.2 ± 1.2
	Glucose (mg/dL)	97.5 ± 14.9
	HbA1C (%)	5.4 ± 0.6
	HDL (mg/dL)	52.8 ± 14.6
	LDL (mg/dL)	124.2 ± 34.8
	TG (mg/dL)	114.9 ± 54.0
	CRP (mg/L)	3.3 ± 3.9

Abbreviations: HTN, hypertension; DM, diabetes mellitus; OSA, obstructive sleep apnea; IHD, ischemic heart disease; CVA, cardiovascular accident; TIA, transient ischemic attack; BMI, body mass index; BP, blood pressure; HR, heart rate; bpm, beats per minute; METS, metabolic equivalents.

**Table 2:** Descriptive statistics of OST measurements in the corneal regions.

Corneal Region	RE Medial Canthus	RE Central Cornea	RE Lateral Canthus	LE Medial Canthus	LE Central Cornea	LE Lateral Canthus
Average temperature, °C ± SD (range)	30.7 ± 1.1 (26.9–33.0)	29.7 ± 1.2 (25.7–32.0)	30.2 ± 1.1 (25.9–32.6)	31.2 ± 1.1 (27.3–34.8)	29.8 ± 1.2 (25.6–34.0)	29.8 ± 1.2 (25.9–33.7)

Abbreviations: RE, right eye; LE, left eye.

**Table 3:** OST differences in the presence of cardiovascular risk factors and cardiovascular disease.

Variable (*n*)	LE Medial Canthus		LE Cornea		LE Lateral Canthus	
Sex		*p* = 0.12		*p* = 0.01 *		*p* = 0.15
Males (88)	31.3 ± 1.2		30 ± 1.2		29.9 ± 1.2	
Females (55)	31 ± 1.1		29.5 ± 1.2		29.7 ± 1.2	
IHD		*p* = 0.02 *		*p* = 0.02 *		*p* = 0.03 *
No (131)	31.1 ± 1.1		29.7 ± 1.2		29.8 ± 1.2	
Yes (8)	32.1 ± 0.9		30.8 ± 0.9		30.7 ± 0.9	
HTN		*p* = 0.4		*p* = 0.3		*p* = 0.3
No (118)	31.1 ± 1.2		29.7 ± 1.3		29.8 ± 1.2	
Yes (21)	31.4.1 ± 1.1		30 ± 1.1		30.1 ± 1	
DM		*p*= 0.4		*p*= 0.6		*p* = 0.8
No (127)	31.2 ± 1.1		29.8 ± 1.2		29.8 ± 1.2	
Yes (12)	31.4 ± 1.3		30.1 ± 1.1.3		30.1 ± 1.3	
Active smoking		*p* = 0.3		*p* = 0.2		*p* = 0.2
No (114)	31.2 ± 1.2		29.8 ± 1.3		29.9 ± 1.2	
Yes (24)	30.9 ± 0.9		29.5 ± 0.9		29.6 ± 1	
Dyslipidemia		*p* = 0.3		*p* = 0.1		*p* = 0.1
No (68)	31.1 ± 1.1		29.6 ± 1.1		29.7 ± 1.1	
Yes (71)	31.3 ± 1.2		30 ± 1.3		30 ± 1.2	
Physical activity		*p* = 0.4		*p* = 0.2		*p* = 0.3
No (19)	31.3 ± 1.2		29.9 ± 1.2		29.9 ± 1.1	
Yes (114)	31.1 ± 1.1		29.7 ± 1.3		29.8 ± 1.2	
Anemia		*p* = 0.2		*p* = 0.2		*p* = 0.3
No (129)	31.1 ± 1.2		29.8 ± 1.2		29.8 ± 1.2	
Yes (9)	31.3 ± 1		30 ± 1.1		30 ± 1	
OSA		*p* = 0.3		*p* = 0.3		*p* = 0.4
No (135)	31.2 ± 1.2		29.8 ± 1.2		29.8 ± 1.2	
Yes (4)	31.8 ± 0.6		30.5 ± 0.6		30.3 ± 0.5	
Normal CST result		*p* = 0.8		*p* = 0.6		*p* = 0.7
No (13)	31.2 ± 1.2		29.9 ± 1.3		29.9 ± 1.2	
Yes (119)	31.1 ± 1.1		29.7 ± 1.2		29.8 ± 1.2	

* Statistically significant (*p* ≤ 0.05, one way ANOVA). Abbreviations: IHD, ischemic heart disease; DM, diabetes mellitus; HTN, hypertension; OSA, obstructive sleep apnea; CST, cardiac stress test.

**Table 4:** Patient and environmental characteristics correlations with OST.

	Medial Canthus		Central Cornea		Lateral Canthus	
**Patient Characteristics**						
Age (years)	*r* = −0.2	*p* = 0.02 *	*r* = −0.17	*p* = 0.047 *	*r* = −0.15	*p* = 0.09 **
Body temperature (°C)	*r* = 0.15	*p* = 0.07 **	*r* = 0.13	*p* = 0.13	*r* = 0.18	*p* = 0.04 *
BMI	*r* = −0.04	*p* = 0.68	*r* = −0.006	*p* = 0.95	*r* = −0.03	*p* = 0.7
Systolic BP (mmHg)	*r* = −0.02	*p* = 0.79	*r* = 0.04	*p* = 0.66	*r* = −0.04	*p* = 0.67
Maximal HR (bpm)	*r* = 0.29	*p* = <0.01 *	*r* = 0.27	*p* < 0.01 *	*r* = 0.27	*p* < 0.01 *
LDL (mg/dL)	*r* = 0.22	*p* < 0.01 *	*r* = 0.24	*p* < 0.01 *	*r* = 0.2	*p* = 0.02 *
HDL (mg/dL)	*r* = −0.18	*p* = 0.03 *	*r* = −0.21	*p* = 0.01 *	*r* = −0.16	*p* = 0.06 **
HbA1C (%)	*r* = 0.03	*p* = 0.73	*r* = 0.08	*p* = 0.32	*r* = 0.06	*p* = 0.47
**Environmental Characteristics**						
Room temperature (°C)	*r* = 0.52	*p* < 0.01 *	*r* = 0.5	*p* < 0.01 *	*r* = 0.52	*p* < 0.01 *
Humidity (%)	*r* = 0.36	*p* < 0.01 *	*r* = 0.38	*p* < 0.01 *	*r* = 0.38	*p* < 0.01 *

Pearson correlation coefficients with respective *p* values are presented. * Statistically significant (*p* ≤ 0.05, 2-tailed), ** Borderline statistical significance (0.05 ≤ *p* ≤ 0.1, 2-tailed). Abbreviations: BMI, body mass index; BP, blood pressure; HR, heart rate; bpm, beats per minute.

**Table 5:** The effect of clinical and environmental factors on OST at the different ocular surface regions.

Dependent Variable: OST at the Medial Canthal Region (R² = 0.5)			
Variable	Unstandardized Coefficients (B)	Standardized Coefficients (Beta)	*p*-Value
Room temperature (°C)	0.32	0.48	<0.01
Maximal HR (bpm)	0.02	0.25	<0.01
IHD	1.31	0.21	<0.01
Body temperature (°C)	1.02	0.19	0.02
LDL (mg/dL)	0.01	0.17	0.04
**Dependent variable: OST at the central corneal region (R² = 0.5)**			
**Variable**	**B**	**Beta**	***p*-Value**
Room temperature (°C)	0.34	0.51	<0.01
Maximal HR (bpm)	0.02	0.29	<0.01
IHD	1.48	0.24	<0.01
**Dependent variable: OST at the lateral canthal region (R² = 0.5)**			
**Variable**	**B**	**Beta**	***p*-Value**
Room temperature (°C)	0.31	0.48	0.01
Maximal HR (bpm)	0.02	0.22	<0.01
IHD	1.30	0.22	<0.01
Body temperature (°C)	1.01	0.2	0.02
LDL (mg/dL)	0.01	0.18	<0.01

Multiple linear regression models stepwise method are presented. Abbreviations: IHD, ischemic heart disease; HR, heart rate.
